# VCAM-1 as a common biomarker in inflammatory bowel disease and colorectal cancer: unveiling the dual anti-inflammatory and anti-cancer capacities of anti-VCAM-1 therapies

**DOI:** 10.1007/s10555-025-10258-2

**Published:** 2025-03-17

**Authors:** Jessica R. Pickett, Yuao Wu, Hang Thu Ta

**Affiliations:** School of Environment and Science, Griffith University, Nathan Campus, Brisbane, 4111 QLD Australia

**Keywords:** Colorectal disease, Inflammatory bowel disease, Colorectal cancer, Inflammation, Vascular cell adhesion molecule-1, Anti-VCAM-1 therapy

## Abstract

Vascular cell adhesion molecule (VCAM)-1 has garnered significant research attention due to its potential as a disease biomarker and drug target across several inflammatory pathologies—including atherosclerosis, asthma, rheumatoid arthritis, and inflammatory bowel disease (IBD). The VCAM-1 protein has also been noted for its functional involvement in cancer metastasis and drug resistance to conventional chemotherapeutics. Although the anti-inflammatory and anti-cancer facets of VCAM-1 antagonisation have been examined separately, there is yet to be a review that explicitly addresses the functional interrelationship between these mechanisms. Furthermore, the pleiotropic mechanisms of anti-VCAM-1 therapies may present a useful paradigm for designing drug candidates with synergistic anti-inflammatory and anti-tumorigenic effects. The pathological overlap between inflammatory bowel disease (IBD) and colitis-associated colorectal cancer (CRC) serves as the quintessential disease model to observe this therapeutic duality. This review thereby details the adhesive mechanisms of VCAM-1 in colorectal disease—specifically, driving immune cell infiltration during IBD and tumour cell metastasis in CRC—and posits the potential of this receptor as a common drug target for both diseases. To explore this hypothesis, the current progress of novel VCAM-1-directed drug candidates in experimental models of IBD and CRC is also discussed.

**Graphical Abstract:**

**TOC Figure:** Graphical abstract illustrating the multi-functional role of vascular cell adhesion molecule (VCAM)-1 in colorectal diseases. VCAM-1 facilitates adhesive cell-to-cell attachments *via* a receptor-ligand binding mechanism with its complementary integrin ligands, α_4_β_1_ and α_4_β_7_. These VCAM-1-mediated interactions are involved in both inflammatory cell recruitment during inflammatory bowel disease (IBD) and cancer cell metastasis in colorectal cancer (CRC), highlighting the therapeutic potential of VCAM-1 as a drug target for both pathologies

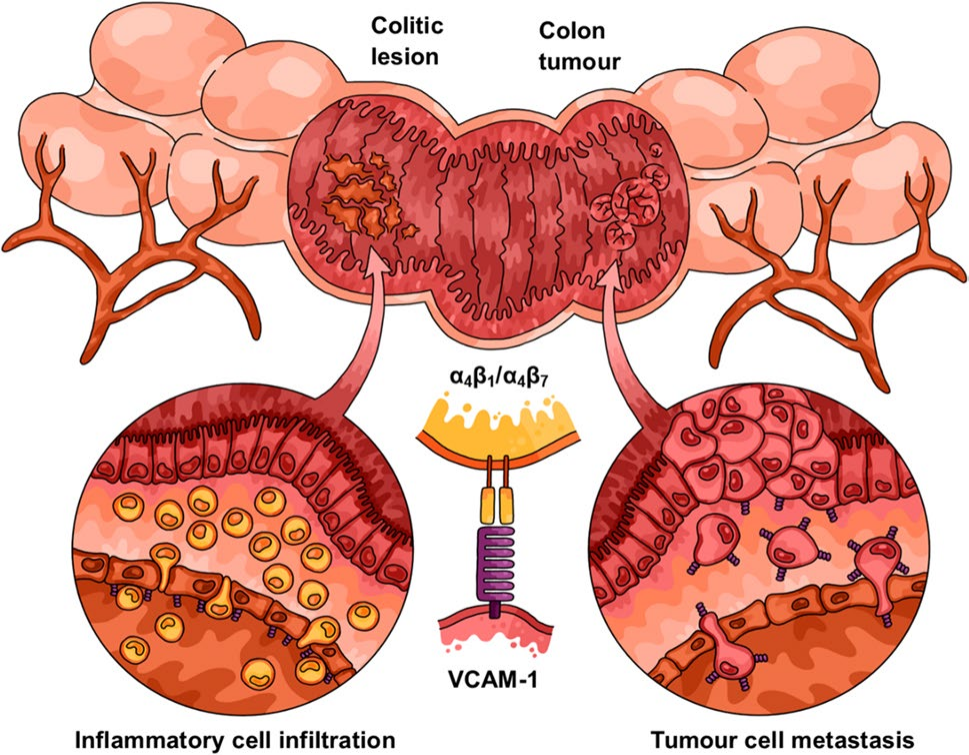

## Introduction

Inflammation has long been established as one of the classical hallmarks of cancer [1]. One of the most extensively researched examples of the predisposition of chronic inflammation to malignant transformation is the marked risk association between inflammatory bowel disease (IBD) and colitis-associated colorectal cancer (CRC) [2–4]. Colitis-associated CRC generally exhibits more advanced staging and aggressive clinical course compared to the sporadic and hereditary forms of the disease [2]. However, the lack of clarity surrounding the molecular mechanisms driving the transition from intestinal inflammation to dysplasia means there is yet to be a definitive chemopreventive agent for colitis-associated CRC in clinical practise [3]. Vascular cell adhesion molecule (VCAM)−1 has shown remarkable promise as a biomarker and drug target across several autoimmune and cancerous pathologies [5]. Existing reviews have reported the therapeutic effects of VCAM-1 in inflammation and cancer separately [5, 6], but this is the first to definitively explore the common roles of VCAM-1 across both disease contexts. Colitis-associated CRC presents the ideal disease model for observing the potential synergy of the distinct anti-inflammatory and anti-metastatic mechanisms of VCAM-1. Despite this, a striking research gap remains in exploring the effects of anti-VCAM-1 therapies in colitis-associated colorectal cancer or as a chemopreventive agent in IBD. To evaluate the practicality of targeting VCAM-1 in colorectal diseases, this review discusses the recent progression of novel VCAM-1-directed therapeutics and their efficacy in clinical trials and experimental models of IBD and CRC.

## Linking colorectal inflammation and tumorigenesis

### Inflammatory bowel disease and colitis

IBD refers to a group of idiopathic, chronic, and relapsing inflammatory conditions affecting the gastrointestinal (GI) tract, with the two archetypal phenotypes being Crohn’s disease (CD) and ulcerative colitis (UC) [7]. Although the etiology of IBD remains elusive, it is generally agreed that it results from an aberrant and dysregulated immune response against the gut microbiota [8]. This state of sustained inflammation in the intestinal mucosa presents clinically as episodes of abdominal pain, diarrhoea, bloody stools, and weight loss, which may eventually culminate in long-term or even irreversible damage to the GI mucosal tissue [7, 9]. Despite continuing research into new treatment options and strategies, the current health outcomes for IBD are far from optimal, with patients constantly shifting between remission and relapse status [8]. Moreover, the risk of neoplastic progression from chronic inflammation to malignancy means that colitis-associated CRC is a life-threatening complication that accounts for about 10–15% of IBD-associated mortality [4]. Another significant concern is the proportion of IBD patients who either do not respond to or gradually develop a resistance to conventional immunomodulators over long-term treatment regimens [10]. These challenges highlight the need to commercialise new and alternative drugs to expand the arsenal of existing treatment strategies available to severe and refractory IBD patients.

Although several immunological mechanisms contribute to the pathogenesis of IBD, disease progression is driven largely by the dysregulated trafficking of immune cells, particularly T cells, to the gut. Even under normal physiological conditions, the colonic mucosa contains a large amount of mononuclear infiltrate due to its constant exposure to dietary antigens, commensal microbes, and pathogens in the intestinal lumen [11]. Tissue-resident memory CD4^+^ T cells are essential for antigen-specific recognition and the consequent adaptive immune response during infection [12]. This resident T-cell population, therefore, needs to be maintained at a level sufficient for protective immunity against harmful pathogens without inducing autoimmunity to normal gut microbiota and innocuous food antigens [13]. The homeostatic balance of T-cell recruitment and retention within the gut is dictated predominately by cell adhesion molecules (CAMs)—a collection of endothelial surface receptors that work together to mediate the sequential rolling, adhesion, and transmigration of leukocytes across the intestinal epithelium [14]. Initial leukocyte rolling results from the concurrent formation and dissociation of selectin-ligand and low-affinity integrin-ligand interactions between circulating T cells and the intestinal endothelium [15]. These “tethering” interactions, while not strong enough for firm cell adhesion or arrest, expose rolling T cells to chemotactic mediators, prompting the molecular switch from selectin-mediated rolling to high-affinity integrin-mediated cell recruitment [14].

Integrin-mediated cell recruitment is a critical step in the tissue-specific homing of T cells to the colonic mucosa, specifically by mediating the transition from leukocyte tethering to arrest and recruitment across the intestinal epithelium. In their quiescent state, circulating T cells express α_L_β_2_, α_4_β_7_, and α_4_β_1_ integrins in an inactivated, low-affinity conformation [16]. However, chemokine-dependent inflammatory activation during leukocyte rolling induces a conformational change from low- to high-affinity integrins, which readily form firm adhesions with their endothelial counter-ligands, intercellular adhesion molecule (ICAM)−1 and ICAM-2, mucosal addressin cell adhesion molecule (MAdCAM)−1, and VCAM-1 [15]. Arrested T cells then polarise and transmigrate across the intestinal epithelium into the colonic mucosa, where they proliferate and differentiate to perform their respective effector functions in maintaining gut immunohomeostasis [17]. Excessive infiltration of the lamina propria by CD4^+^ T cells, however, is a hallmark feature of chronic intestinal inflammation and IBD [13]. Through pro-inflammatory signalling, the overabundant T-cell population promotes inflammatory phenotypes of other innate cells—including epithelial cells, phagocytes, and myofibroblasts—thereby fuelling self-perpetuating hyperresponsiveness to innocuous antigens in the intestinal lumen [16]. This T-cell-driven autoimmunity results in a state of chronic intestinal inflammation and colonic tissue damage, clinically manifesting as IBD.

### Colorectal cancer

CRC is the third-most common form of malignancy and the fourth-most common cause of cancer-related death worldwide [18]. Tumorigenesis initiates in the normal intestinal mucosa, usually developing from adenomatous polyps that eventually infiltrate the submucosa and metastasise if left untreated [19]. As is the general principle of most cancers, CRC is typically a sporadic disease driven by somatic mutations; however, it may also result as a complication of long-term colonic inflammation, such as in IBD [20]. Indeed, the risk of developing CRC is 1.5- to 2.4-fold higher among IBD patients compared to the general population [21]. Currently, the first line of defence against CRC is early detection and treatment of precursor lesions through faecal occult blood testing and colonoscopy surveillance [22]. The prognostic significance of inflammatory biomarkers in CRC [23] and the beneficial effects of anti-inflammatory drugs in CRC chemoprevention [24] suggest a potential utility for anti-inflammatory novel therapeutics targeting both sporadic and colitis-associated CRC. As such, studies in novel immunotherapies targeting tumorigenesis are rapidly gaining traction, with the goal of not only reducing CRC incidence and mortality but also improving clinical outcomes for patients with advanced and refractory diseases that may escape early surveillance procedures.

The current understanding of CRC progression is that neoplastic lesions originate as aberrant crypt foci, which may subsequently develop into adenomatous polyps and, ultimately, colorectal carcinoma [25]. The most well-understood underlying mechanism that drives the colorectal adenoma-to-carcinoma sequence is the accumulation of genetic and epigenetic mutations in oncogenes and tumour suppressor genes, which is defined as sporadic CRC [26]. However, colitis-associated CRC demonstrates a molecularly distinct, accelerated version of this sequence stemming from inflammatory gene expression and tissue damage [27]. While sporadic mutations tend to result from chromosomal and microsatellite instability, inflammation-induced mutations are generally attributed to oxidative DNA damage by reactive oxygen species (ROS) [2]. Furthermore, the carcinogenic effects of inflammatory signalling pathways have also been observed, including, but not limited to, immune cell recruitment by tumour necrosis factor-α (TNF-α) and nuclear factor-κB (NF-κB), pro-proliferative and anti-apoptotic effects of interleukin (IL)−16 and IL-22, and epithelial STAT3 signalling [28]. Although better characterised in colitis-associated CRC, in recent years, there has also been a growing body of evidence supporting the role of inflammation in potentiating sporadic colorectal tumours [29]. The inherent relationship between inflammation and carcinogenesis means that anti-inflammatory therapies are a promising avenue for preventing and treating CRC.

## Vascular cell adhesion molecule-1 as a therapeutic target

VCAM-1, or CD106, is a cytokine-inducible surface glycoprotein that mediates leukocyte trafficking to sites of inflammation *via* firm adhesive interactions with its complementary integrin ligands [30]. The receptor is found predominantly on activated endothelial cells of tissue vascular beds, though its expression has also been observed in certain classes of haematopoietic cells and cancer cells [31, 32]. As a member of the immunoglobulin (Ig) superfamily, VCAM-1 structurally consists of six or seven extracellular Ig-like domains, a transmembrane domain, and a cytoplasmic domain [33]. VCAM-1 binds its primary ligand, α_4_β_1_ integrin, *via* IDSPL recognition sequences within its highly homologous first and fourth domains [34]. However, the differential affinities of these two domains create an activation-dependent “molecular switch” dictating VCAM-1-mediated cell recruitment, in which the simultaneous binding of domains one and four necessary for firm adhesion requires prior integrin activation [35]. Additionally, VCAM-1 has demonstrated binding activity with α_4_β_7 _[36], α_M_β_2 _[37], α_9_β_1 _[38], and α_D_β_2 _[39] integrins, although these interactions are not as well defined as that of α_4_β_1_. The firm adhesive interaction between VCAM-1 on activated endothelial cells and high-affinity α_4_β_1_—and, to a lesser extent, α_4_β_7_—on circulating immune cells plays an integral role in driving the transition from initial cell capture and rolling on the endothelium to arrest and transmigration across the vessel wall during inflammation [33].

**Fig. 1:**
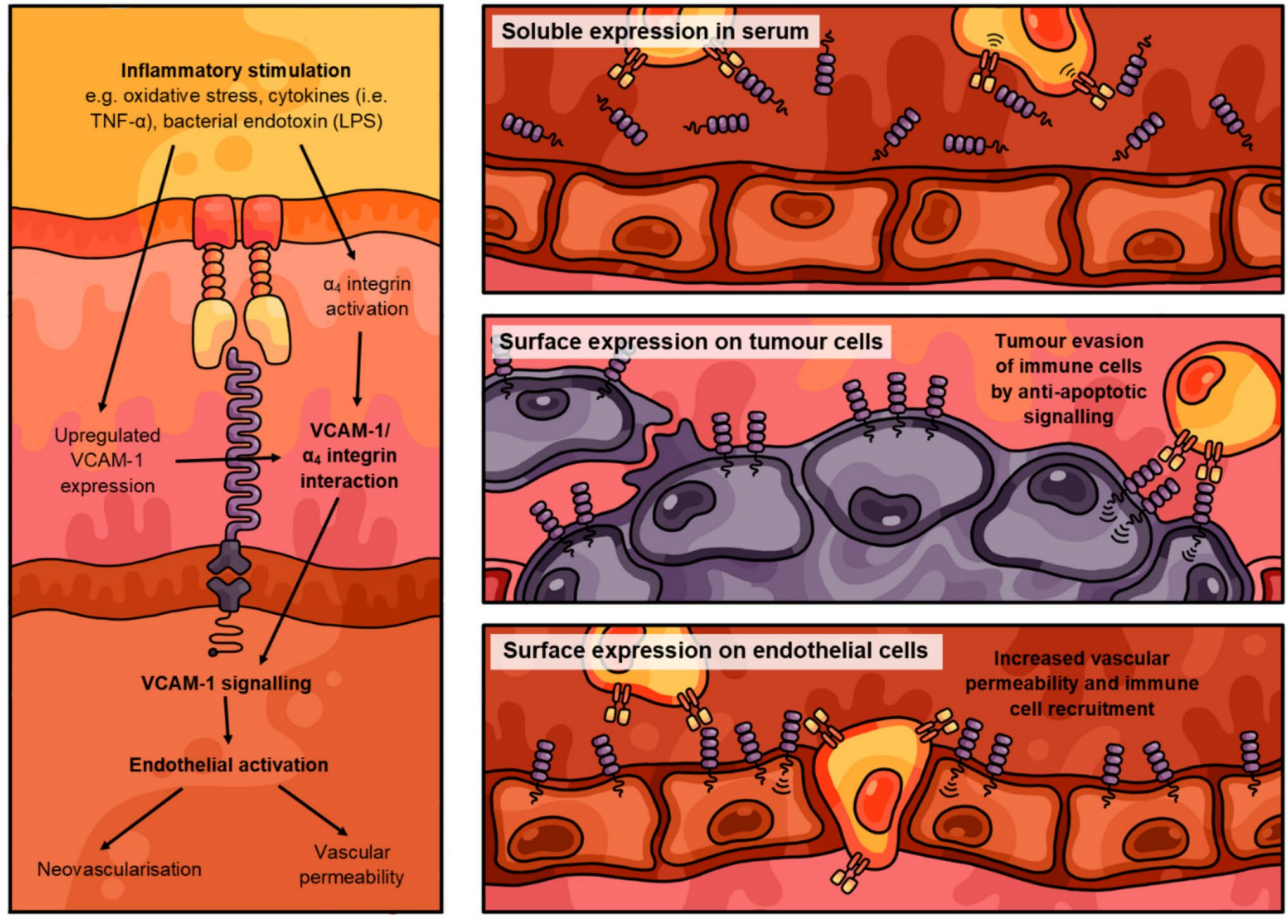
Conceptual diagram illustrating the role of vascular cell adhesion molecule (VCAM)−1 expression and signalling across various tissues. As a common mechanism, surface VCAM-1 expression on affected cells is upregulated by various inflammatory stimuli, after which the protein binds to its corresponding α_4_ integrin ligands on complementary cells. VCAM-1 activation and signalling pathways exert different effects in different cell types. Activated cells shed soluble VCAM-1, which binds to circulating leukocytes in the blood to shape the immune response. Surface VCAM-1 on cancer cells binds tumour-associated macrophages to send anti-apoptotic signals and block tumour cell killing by the immune system. Surface VCAM-1 on inflamed vascular endothelial cells binds to circulating leukocytes in the bloodstream to increase vascular permeability and leukocyte recruitment into adjacent tissues

Aside from its role in mediating leukocyte recruitment, VCAM-1 is also a common molecular component of multiple endothelial signalling pathways—most notably, those involved in regulating vascular permeability and neovascularisation (Fig. 1) [33, 40]. Clustering VCAM-1/α_4_β_1_ and VCAM-1/α_4_β_7_ interactions during inflammatory cell recruitment relay multiple intracellular signals that prompt the cytoskeletal and extracellular remodelling of activated endothelial cells to their inflamed, pathological phenotype [41]. These pathways include a Rac1-mediated signalling cascade that destabilises local adherens junctions between adjacent endothelial cells *via* the clathrin-dependent internalisation of VE-cadherin and redox-driven activation of matrix metalloproteinases [33]. Cells more readily migrate through the weakened endothelial cell-to-cell contacts, resulting in the compromised endothelial barrier and excessive cell infiltration commonly observed in inflammatory and cancerous disease pathologies [41]. Several VCAM-1-activated signalling intermediates involved in regulating vascular permeability—particularly extracellular signal–related kinase (ERK)1/2, mitogen-associated protein (MAP) kinase, and focal adhesion kinase (FAK)—also serve as mediators of angiogenesis [42]. More recently, Kaur et al [40] also found that surface VCAM-1 expression regulates pro-angiogenic IL-8 activation on retinal endothelial cells through the JunB transcription factor to stimulate vessel sprouting and formation. The pleiotropic effects of VCAM-1 in endothelial signalling mean that VCAM-1 activation is an essential step in several molecular mechanisms underlying endothelial dysfunction and, therefore, in a wide range of disease processes involving the vasculature.

The inducible and localised nature of VCAM-1 expression has marked it as a protein of interest across disease pathologies as a predictive disease biomarker and a potential therapeutic target. While VCAM-1 is minimally expressed on most resting vascular endothelial beds, it is significantly upregulated in areas of endothelial dysfunction following injury or stress [31]. Endothelial induction of VCAM-1 expression has been demonstrated following stimulation by various pro-inflammatory factors, including lipopolysaccharide (LPS) [43], TNF-α [44], and several ILs [45–47]. As such, concentrated regions of aberrant VCAM-1 overexpression generally delineate areas of endothelial dysfunction and disease activity in experimental disease models and human IBD patients. Elevated soluble VCAM-1 in the serum and endothelial VCAM-1 expression on the colonic mucosa and associated microvasculature are also considered credible biomarkers for colorectal inflammation [48] and carcinogenesis [49]. Moreover, VCAM-1, along with other CAMs, has been implicated to have a functional role not only in leukocyte trafficking during IBD [50] but also in tumour cell invasion and metastasis in CRC [51]. Naturally, therapeutic blockade of inflammatory cell infiltration and cancer cell metastasis *via* VCAM-1 has been posed as an intriguing strategy for a range of inflammatory and cancerous pathologies, including colorectal diseases.

### Role of VCAM-1 in intestinal inflammation

**Fig. 2:**
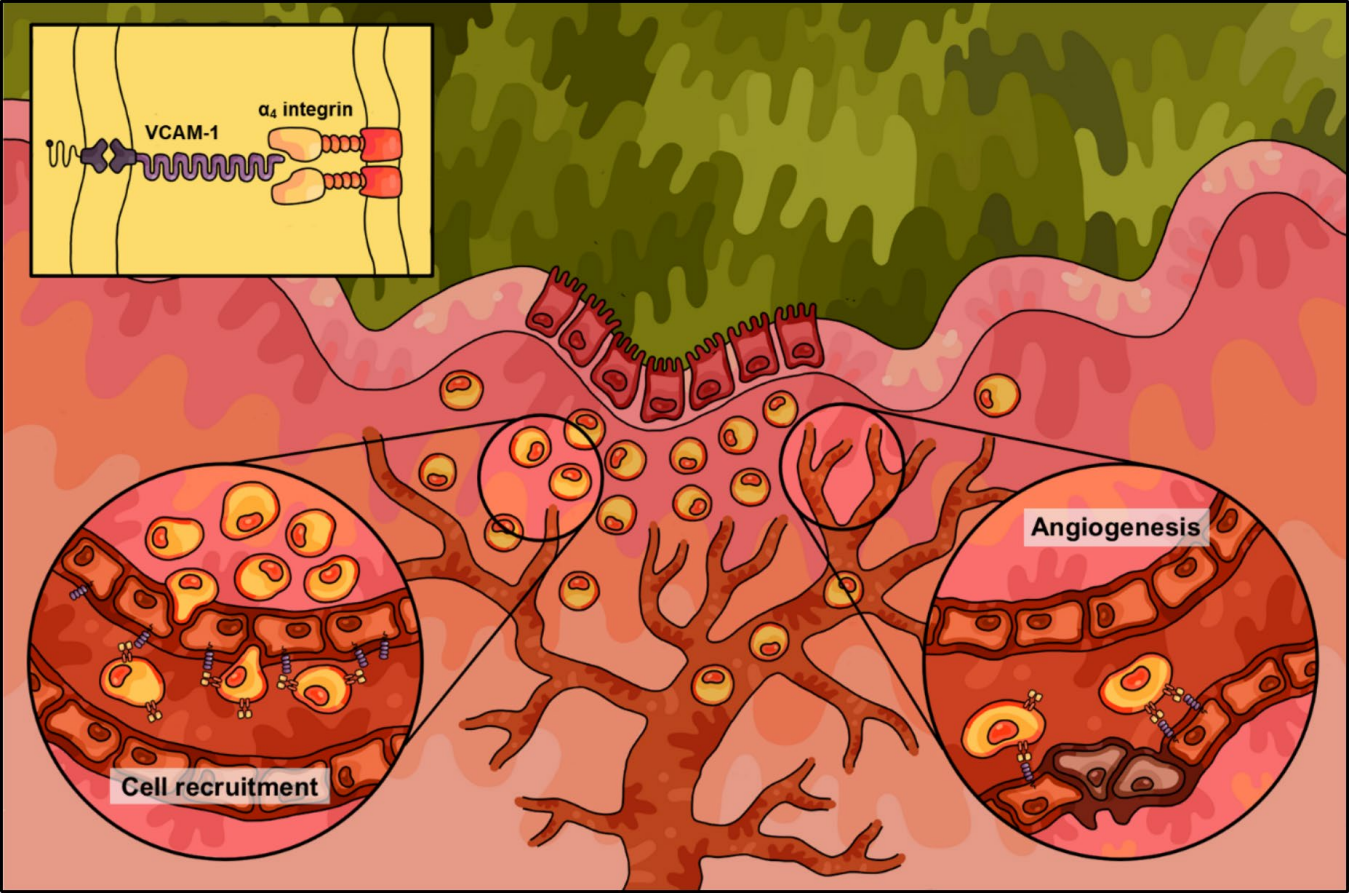
Pictorial overview summarising the mechanisms of vascular cell adhesion molecule (VCAM)−1 in the pathogenesis of inflammatory bowel disease (IBD). Inflamed intestinal microvessels associated with colitic lesions express elevated levels of VCAM-1 compared to the normal vasculature of healthy colon tissues. Surface VCAM-1 receptors bind to complimentary α_4_ integrin ligands to facilitate pathological cell-to-cell adhesions involved in vascular inflammation and other related disease processes. These VCAM-1/α_4_ integrin interactions facilitate leukocyte-endothelial interactions and endothelial signalling pathways involved in inflammatory cell recruitment and angiogenesis, two pathological mechanisms that are critical to IBD

The functional role of VCAM-1 in mediating immune cell trafficking to the inflamed colonic mucosa has established the protein as a central contributor to the pathogenesis of IBD. VCAM-1 involvement in pathological cell recruitment has been defined across a range of inflammatory disease pathologies, including atherosclerosis [52], asthma [53], rheumatoid arthritis [54], and colitis [50]. Several clinical studies have investigated the utility of soluble VCAM-1 as a serum biomarker of colitis, with elevated soluble VCAM-1 levels observed in CD and UC patients. In addition, upregulated VCAM-1 on the activated colonic endothelium during inflammation increases the recruitment of α_4_ integrin-expressing immune cells, contributing to excessive immune cell infiltrate and the dysregulated immune response (Fig. 2). Sans et al [50]. demonstrated the functional significance of VCAM-1 in IBD by observing VCAM-1 upregulation and the consequent increase in leukocyte-endothelial adhesion on colonic venules in murine models of experimental colitis. Furthermore, the authors demonstrated that the selective inhibition of VCAM-1 with a neutralising monoclonal antibody (mAb) reduced VCAM-1-mediated leukocyte recruitment and significantly attenuated macroscopic colonic tissue damage and colitis symptoms [50, 55]. The selective blockade of VCAM-1-mediated leukocyte adhesion has, therefore, been suggested as a precise and effective means of inhibiting dysregulated cell infiltration during intestinal inflammation and colitis.

Naturally, the basis of VCAM-1 as an inducible cell adhesion receptor is the most conspicuous molecular mechanism of its pathological involvement in IBD. However, VCAM-1 also serves as a critical component of specific signalling pathways involved in vascular remodelling and intestinal barrier function, which are both essential processes for sustaining chronic colitis [33, 56]. As discussed earlier, VCAM-1 mediates cell recruitment of T cells and progenitor mast cells across the colonic vascular endothelium into the mucosa *via* firm adhesive interactions between endothelial VCAM-1 and complementary α_4_ integrins on these circulating cells (Fig. 2) [57, 58]. Furthermore, the clustering of VCAM-1/α_4_ integrin interactions initiates Rac1 signalling involved in cell-to-cell junctional weakening, resulting in the increased vascular permeability of activated endothelia [59]. As VCAM-1 has been directly implicated in endothelial dysfunction of the blood–brain barrier [60], a similar role for the protein in disrupting the gut-blood barrier could be plausibly linked to the “leaky gut” physiology commonly observed in IBD patients. Additionally, the pro-angiogenic effects of VCAM-1 activity—such as increased B-cell activation and cytokine signalling—stimulate vessel remodelling of the mucosal and submucosal microvascular architecture, which, in turn, exacerbates inflammatory cell recruitment and mucosal tissue destruction during IBD [58, 61].

### Role of VCAM-1 in colorectal tumour development

**Fig. 3:**
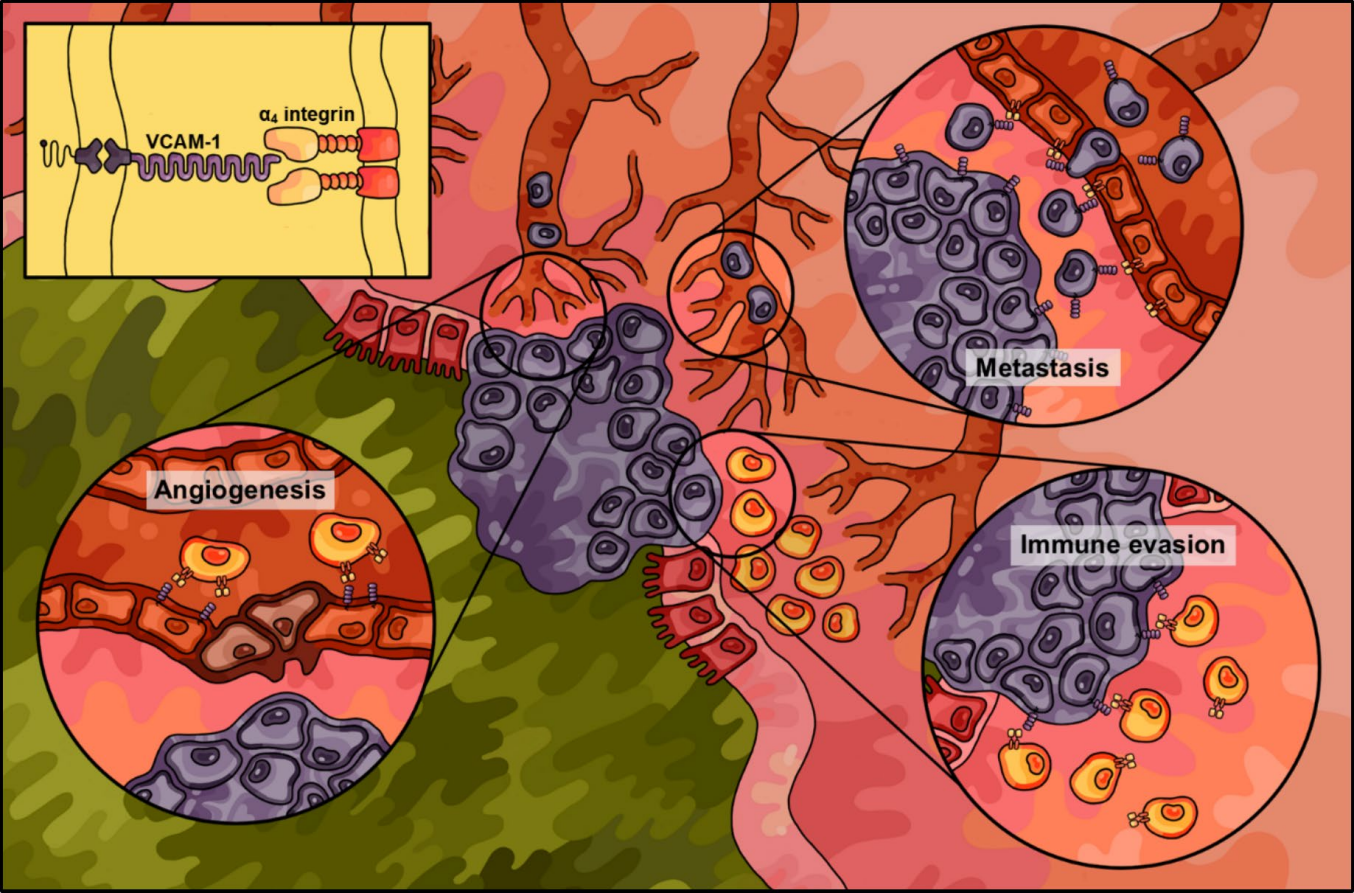
Pictorial overview summarising the mechanisms of vascular cell adhesion molecule (VCAM)−1 in the pathogenesis of colorectal cancer (CRC). Colorectal tumour cells express elevated levels of VCAM-1 compared to healthy colonic epithelial cells. Surface VCAM-1 receptors bind to complimentary α_4_ integrin ligands to facilitate pathological cell-to-cell adhesions involved in vascular inflammation and other related disease processes. These VCAM-1/α_4_ integrin interactions facilitate leukocyte-endothelial, tumour-leukocyte, and tumour-endothelial interactions involved in angiogenesis, immune evasion, and metastasis mechanisms involved in CRC

In addition to its involvement in inflammatory cell trafficking during colitis and IBD, VCAM-1 has recently been implicated to play a critical role in tumour development in CRC *via* similar cell migration–based mechanisms [51]. VCAM-1 overexpression has been reported in several cancer pathologies—including breast [62, 63], ovarian [64, 65], gastric [66, 67], and pancreatic cancer [68, 69]. Studies investigating CRC progression have found a correlation between elevated VCAM-1 expression levels and the extent of tumour development, lymph node involvement, and cancer metastasis [49, 51, 70, 71]. Similar to its adhesive function in immune cell trafficking, VCAM-1 expressed on the surface of tumour cells tethers leukocytes, endothelial cells, and other innate cells that express α_4_ integrins, enhancing adhesion-based processes such as cell invasion and metastasis (Fig. 3) [62]. Indeed, in a study characterising the tumorigenic capabilities of VCAM-1 in CRC, Zhang et al [51]. demonstrated that VCAM-1-overexpressing Caco-2 and RKO colon carcinoma cell lines displayed significantly enhanced cell migration and invasion *in vitro* and pulmonary and hepatic metastasis *in vivo* compared to control and VCAM-1-knockdown lines. In validating the functional role of VCAM-1 in colorectal tumour development and metastasis, VCAM-1-directed therapeutics have been revealed as a potential therapeutic strategy for treating and preventing CRC.

Much like in IBD, the pathogenic contributions of VCAM-1 in CRC are not limited purely to its ability to bind integrin for cell-to-cell adhesion, but also in downstream signalling pathways involved in endothelial dysfunction and tumour cell survival. During CRC, VCAM-1 expression is upregulated both on colorectal tumour cells and endothelial cells of the surrounding microvasculature, therefore playing several distinct functions in cancer development. Though VCAM-1 is generally described as an endothelial protein, several varieties of transformed cancer cells also aberrantly overexpress VCAM-1 on their surface [72]. In a mechanism resembling VCAM-1-mediated leukocyte-endothelial cell attachment, VCAM-1-expressing tumour cells have previously demonstrated a tendency to adhere to cognate α_4_-expressing leukocytes, such as monocytes and tumour-associated macrophages [62, 73]. These leukocytes appear to promote the growth and survival of VCAM-positive tumours and metastases, specifically by conveying anti-apoptotic signals *via* surface VCAM-1 clustering, subsequent recruitment of phosphorylated ezrin, and downstream PI3-kinase/Akt activation [62]. Endothelial VCAM-1 expression on tumour-associated vasculature also plays a significant role in neovascularisation. A study by Ding et al [66] demonstrated that VCAM-1/α_4_ integrin interactions between endothelial cells and pericytes stimulated endothelial sprouting and angiogenesis in gastric cancer. Considering the mounting evidence of VCAM-1 involvement in angiogenesis across several cancer pathologies, it would be expected that aberrant VCAM-1 expression would serve a similar function in CRC.

### Summarising the functional overlap of VCAM-1 in IBD and CRC

As inflammation is one of the classical hallmarks of cancer pathogenesis, it is only natural that certain autoimmune disorders and cancer pathologies share several inflammatory markers and molecular mechanisms [1]. VCAM-1 serves a functional role across several distinct intercellular interactions and signalling pathways—not only as a surface receptor on endothelial cells, but also ectopically in tumour cells and as a soluble form in serum [30, 31]. As such, the concept of VCAM-1 as a functional bridge linking IBD and CRC hinges on its multi-functional involvement in several disease processes underlying colonic inflammation and tumorigenesis. VCAM-1-mediated cell adhesion and transmigration is a well-established functional overlap, facilitating immune cell recruitment in IBD and tumour cell metastasis in CRC [50, 51]. The primary ligand for VCAM-1, α_4_β_1_ integrin, is expressed on multiple cell types—including monocytes, lymphocytes, and many tumour cell types. Therefore, VCAM-1/α_4_β_1_ interactions are remarkably versatile, being capable of leukocyte-endothelial, tumour-leukocyte, and tumour-endothelial attachment [32]. Furthermore, the regulatory effect of VCAM-1 activation and cross-linking on multiple signalling pathways—including vascular permeability [33], angiogenesis [42, 74], and anti-apoptosis [62]—also explains the broad involvement of the protein in both IBD and CRC despite the pathophysiological differences between the two diseases.

Considering the synergistic effects of VCAM-1 in IBD and CRC *via* its multiple cell-to-cell signalling mechanisms, the potential of the protein as a multi-functional drug target is an intriguing platform for precision nanomedicines in colorectal disease treatment. The remainder of this review goes on to summarise the existing evidence and current developments supporting the anti-colitic and anti-cancer potential of several approved and novel therapeutics that target VCAM-1 in colorectal pathologies (listed in Tables 1, 2, and 3).

**Table 1:** List of therapeutic agents used for treating experimental models of inflammatory bowel disease (IBD) that target vascular cell adhesion molecule (VCAM)−1-mediated interactions

Therapeutic	*In vivo* models	Clinical studies	Key findings	Source(s)
**Class**	Anti-integrin monoclonal antibodies (mAbs)			
Natalizumab	N/A	Randomised, double-blind, placebo-controlled trial of patients with active Crohn’s disease (CD) (*n* = 30)	Natalizumab treatment was well-tolerated in CD patients. Remission occurred at a greater proportion with natalizumab compared to placebo, but not to a statistically significant extent	Gordon et al. (2001) [84]
	N/A	Clinical intervention study of patients with active ulcerative colitis (UC) (*n* = 10)	Natalizumab treatment was well-tolerated in UC patients and demonstrated significant improvements in disease symptoms compared to those observed prior to commencing treatment	Gordon et al. (2002) [85]
	N/A	Randomised, double-blind, placebo-controlled trial of patients with active CD (*n* = 248)	Natalizumab treatment was well-tolerated in CD patients. Clinical response rates were significantly higher in natalizumab-treated patients compared to placebo, but there was no statistical difference in clinical remission rates	Ghosh et al. (2003) [86]
	N/A	ENACT-1: Randomised, double-blind, placebo-controlled trial of patients with active CD (*n* = 905) ENACT-2: Randomised, double-blind, placebo-controlled trials of patients with active Crohn’s disease (*n* = 339)	Natalizumab treatment was well-tolerated in CD patients In the first trial (ENACT-1), natalizumab induction therapy resulted in small, non-significant improvements in response and remission rates. Patients who had response in the initial trial had significantly increased rates of sustained response and remission in a second, follow-up trial (ENACT-2)	Sandborn et al. (2005) [87]
	N/A	Randomised, double-blind, placebo-controlled trial of patients with moderate-to-severely active CD (*n* = 509)	Natalizumab treatment was relatively well-tolerated in CD patients, and adverse events occurred at similar frequencies in the natalizumab and placebo groups. Natalizumab-treated individuals had significantly higher rates of clinical remission compared to those receiving placebo	Targan et al. (2007) [88]
	N/A	Randomised, double-blind, placebo-controlled of patients with active CD currently receiving infliximab (*n* = 79)	Natalizumab treatment was relatively well-tolerated in CD patients. Natalizumab treatment in conjunction with infliximab significantly attenuated disease activity in comparison with infliximab alone	Sands et al. (2007) [89]
	N/A	Retrospective case review of patients with active Crohn’s disease (*n* = 49)	Natalizumab treatment was relatively well-tolerated in CD patients. Natalizumab induction therapy increased induction and maintenance of patients with CD	Sakuraba et al. (2013) [90]
	N/A	Retrospective case review of pediatric CD patients receiving natalizumab treatment who had previously failed TNF-α therapies (*n* = 9)	Natalizumab treatment was well-tolerated and induced both clinical response and remission in multiple patients. No serious adverse effects were observed	Singh et al. (2016) [92]
**Class**	Anti-integrin small molecule drugs (SMDs)			
AJM300	N/A	Randomised, double-blind, placebo-controlled Phase IIa study of patients with moderately active UC (*n* = 102)	AJM300 treatment was well-tolerated in UC patients. AJM300 significantly increased clinical response and remission rates and mucosal healing compared to placebo control. No serious adverse events, including progressive multifocal leukoencephalopathy, were observed	Yoshimura et al. (2015) [100]
	N/A	Randomised, double-blind, placebo-controlled Phase III study of patients with UC	AJM300 treatment was well-tolerated in UC patients, and adverse events occurred at similar frequencies in the AJM300 and placebo groups. AJM300-treated individuals had significantly higher rates of clinical response compared to those receiving placebo	Matsuoka et al. (2022) [97]
**Class**	Anti-VCAM-1 monoclonal antibodies (mAbs)			
5F10	TNBS-induced colitis in male Sprague–Dawley rats	N/A	5F10 treatment significantly decreased leukocyte rolling and adhesion in colitic rats to levels resembling that of control animals. 5F10 also significantly improved colitis symptoms *in vivo*	Sans et al. (1999) [50]
MK2.7	SAMP/Yit mice and adoptive transfer of CD4^+^ T-cells from SAMP/Yit mice into SCID mice	N/A	MK2.7 treatment alone failed to show significant resolution of colitis symptoms. However, MK2.7 in combination with YN-1 (anti-ICAM-1 mAb) demonstrated significant therapeutic benefit in active, but not chronic, inflammation	Burns et al. (2001) [78]
MK1.91	DSS-induced colitis in male CD_1_ mice	N/A	MK1.91 treatment decreased leukocyte rolling and adhesion in colitic models to levels comparable to that of control animals. MK1.91 also significantly attenuated colitis symptoms and intestinal inflammation	Soriano et al. (2000) [55]
429	TNF-α-stimulated colon inflammation in female BALB/c mice	N/A	429 treatment decreased rolling and adhesion of leukocytes on the inflamed colonic and small intestinal mucosa	Watanabe et al. (2002) [80]
**Class**	Anti-VCAM-1 antisense oligonucleotides (ASOs)			
ISIS 18155	Indomethacin-induced ileitis in male Sprague–Dawley rats	N/A	ISIS 18155 treatment significantly decreased VCAM-1 expression on submucosal and mesenteric vessels and reduces leukocyte rolling and adhesion *in vivo*. ISIS 18155 also significantly attenuated macroscopic and histological inflammation	Rijcken et al. (2002) [79]

**Table 2:** List of novel vascular cell adhesion molecule (VCAM)−1-targeting imaging systems employed in experimental models of inflammatory bowel disease (IBD) and colorectal cancer (CRC)

Ligand	Carrier	Label	*In vitro* models	*In vivo* models	Key findings	Source(s)
**Class**	Monoclonal antibodies (mAbs) and single-chain variable fragments (scFvs)					
anti-VCAM-1 mAb (5F10)	None	Iodine-123 (^123^I)	N/A	2,4,6-trinitrobenz enesulphonic acid (TNBS)-induced colitis in male Sprague–Dawley rats	^123^I-5F10 antibodies were effective at visualising colonic inflammation *in vivo*. Scintigraphy uptake was significantly higher in the colon of colitic animals compared to controls	Sans et al. (2001) [121]
anti-VCAM-1 scFv	None	Technetium-99 m (^99m^Tc)	N/A	TNBS-induced colitis in male New Zealand white rabbits	^99m^Tc-anti-VCAM-1 scFvs were effective at visualising colonic inflammation *in vivo*. Scintigraphy uptake was significantly higher in the colon of colitic animals compared to controls. Autoradiography confirmed specific probe accumulation and colitic lesions	Liu et al. (2019) [122]
**Class**	VCAM-1-conjugated microbubbles					
anti-VCAM-1 mAb	Lipid-shelled microbubbles	AF647-conjugated secondary antibody or YOYO-1 labelled plasmid	Laminar flow adhesion assays on recombinant VCAM-1 and SVEC4-10 monolayers	Spontaneous colitis in TNFΔARE mice	Fluorophore-containing, VCAM-1-targeted microbubbles were effective at visualising colonic inflammation *in vitro*. Microbubbles bound to recombinant proteins and endothelial monolayers substrates under flow *in vitro*. Fluorescent *ex vivo* imaging confirmed specific microbubble accumulation at sites of endothelial inflammation along the gastrointestinal (GI) tract	Tlaxca et al. (2013) [123]
anti-VCAM-1 peptide (VHPKQHRG-GSK)	Lipid-shelled microbubbles	Fluorescein isothiocyanate (FITC)	N/A	MC38 murine colon adenocarcinoma tumors injected into leg muscles of C57BL/6 mice	VCAM-1-targeted microbubbles were effective at visualising tumour vasculature *in vivo*. Ultrasound molecular imaging confirmed specific microbubble retention in the tumour vasculature with significantly less accumulation in healthy contralateral muscle	Unnikrishnan et al. (2019) [130]

**Table 3:** List of novel vascular cell adhesion molecule (VCAM-1)-targeting therapeutic systems employed in experimental models of inflammatory bowel disease (IBD) and colorectal cancer (CRC)

Ligand	Carrier	*In vitro* models	*In vivo* models	Key findings	Source(s)
**Class**	Mesenchymal stem cells (MSCs)				
Anti-VCAM-1 mAb	MSCs	N/A	TNBS-induced colitis in female BALB/c mice	Injected V-MSCs selectively homed to the injured colon in colitic mouse models compared to uncoated MSCs. V-MSC treatment also significantly improved mucosal healing and attenuated colon symptoms	Chen et al. (2019) [146]
Anti-VCAM-1 mAb	MSCs	N/A	DSS-induced colitis in C57BL/6 mice	Injected V-MSCs selectively homed to the injured colon in colitic mouse models compared to uncoated MSCs. V-MSC treatment significantly improved colitis symptoms and survival rates in colitic mouse models	Ko et al. (2010) [138]
**Class**	Polymeric nanoparticles (NPs)				
Anti-VCAM-1 mAb (429)	PLA-PEG NPs	N/A	DSS-induced colitis in C57BL/6 J mice	Injected NPs selectively homed to the colonic vasculature in colitic mouse models	Sakhalkar et al. (2005) [143]
**Class**	Microbubbles				
Anti-VCAM-1 peptide	Lipid-shelled microbubbles	N/A	Spontaneous colitis in TNFΔARE mice	Injected microbubbles selectively homed to the colitic vasculature and allowed for specific gene delivery of plasmid for treating colitis symptoms	Tlaxca et al. (2013) [123]

## Therapies that block VCAM-1 binding activity

Although conventional anti-inflammatory therapeutics for IBD and CRC have traditionally been aimed at suppressing pleiotropic inflammatory mediators, the specific focal blockade of VCAM-1-mediated immune cell trafficking has gained considerable attention as a precise and effective strategy against colonic disease. Using non-specific immune modulators to ubiquitously block several molecular signalling pathways, while effective in countering the inflammatory response, may sometimes actually be overeffective in suppressing immune function. This is evidenced by the association of conventional immune suppressors and biologics with increased risks for opportunistic infections and other adverse side effects during treatment for IBD and CRC [75]. On the other side of the coin, the significant incidence of lack or loss of response in patients receiving anti-TNF-α agents seriously undermines the therapeutic efficacy of anti-colitic and anti-cancer drugs that target immune mediators [76]. These observations raise questions as to whether downstream drug targets might be less susceptible to compensation by other inflammatory signalling pathways. By limiting drug activity to VCAM-1-mediated interactions, selective antibody and small molecule drug (SMD) antagonists can, therefore, circumvent the traditional pitfalls associated with broad-spectrum immunomodulators.

The concept of anti-adhesion therapies selectively targeting endothelial CAM receptors has gained considerable traction in colorectal disease research due to their integral role in inflammatory cell recruitment and cancer cell metastasis. Currently, the three primary therapeutic targets for selective immunoblockade of leukocyte recruitment across the colonic epithelium are VCAM-1/α_4_β_1_, ICAM-1/α_2_β_2_, and MAdCAM-1/α_4_β_7 _[77]. Pre-clinical studies targeting different routes of cell trafficking show that selecting the receptor-ligand interaction that most optimally balances efficiency and selectivity is not a simple task. Comparative investigations by Sans et al [50] and Soriano et al [55] evaluating the differential effectiveness of CAM-immunoneutralising antibodies in murine colitis demonstrated that selective blockade of the VCAM-1 axis was superior to that of the ICAM-1 and MAdCAM-1 axes. However, this balance of efficacy is highly variable between different *in vitro* and *in vivo* models of colitis [50, 55, 78–80]. Although all three CAM receptors have been defined as suitable target ligands for colonic disease treatment, VCAM-1 has recently garnered particular interest after being revealed as a critical facilitator of colon tumour survival and metastasis in addition to its established role in intestinal inflammation [51].

### Anti-integrin antibodies

Anti-integrin antibodies have shown a great degree of promise as novel anti-colitic drug candidates in both pre-clinical and clinical studies and, therefore, could pose a viable strategy for blocking VCAM-1/α_4_β_1_-mediated cell recruitment across various colorectal pathologies. Antagonists to the α_4_ subunit of the integrin heterodimer have the distinct advantage of being able to inhibit both α_4_β_1_ and α_4_β_7_, effectively blocking both VCAM-1/α_4_β_1_- and MAdCAM-1/α_4_β_7_-mediated routes of leukocyte extravasation [81]. Natalizumab (Tysabri^©^) is a chimeric recombinant human IgG_4_ anti-α_4_ integrin antibody that has been FDA-approved for treating multiple sclerosis and moderate-to-severe CD [82, 83]. Several clinical studies support the therapeutic potential of natalizumab for inducing and maintaining remission in patients with active IBD [84–91]. Interestingly, natalizumab treatment has demonstrated significant benefit in patients exhibiting a lack or loss of response to TNF-α antagonists, suggesting a potential superiority in drug mechanism compared to conventional biologics [90, 92]. However, the reported association between natalizumab treatment and progressive multifocal leukoencephalopathy, a life-threatening opportunistic infection of the central nervous system, has raised significant concerns regarding its safety profile [93]. Natalizumab use has, therefore, been restricted to adult patients with moderate-to-severely active CD who have previously discontinued conventional therapies or TNF-α antagonists [83].

Despite its therapeutic promise, the initial enthusiasm regarding natalizumab has noticeably dampened in recent years, particularly following the emergence of alternative integrin inhibitors with superior safety profiles. Research attention has shifted primarily towards vedolizumab (Entyvio®), a monoclonal antibody specifically blocking the α_4_β_7_, but not the α_4_β_1_, heterodimer [94]. Following its therapeutic success in clinical trials, vedolizumab has been approved for treating and maintaining moderate-to-severe UC and CD, albeit only in patients with insufficient response to conventional pharmaceuticals [95]. Most importantly, due to the gut-specificity of the MAdCAM-1/α_4_β_1_ interaction, vedolizumab exhibits no observed association with progressive multifocal encephalopathy, unlike natalizumab [94]. Theoretically, natalizumab would appear to be a promising anti-cancer drug due to its ability to block integrin-mediated tumour cell adhesion and migration in metastasis. Although natalizumab treatment has been shown to display inhibitory multiple myeloma development *in vivo* [96], the anti-cancer potential of natalizumab has yet to be definitively established. In more recent years, research attention has been turning away from natalizumab towards more specific inhibitors in favour of safety over a broader spectrum of inhibition. Nonetheless, further clinical investigation and reviews of current literature are warranted to define optimal treatment parameters for natalizumab as a safe and effective IBD treatment.

### Small molecule drug (SMD) integrin inhibitors

SMD integrin inhibitors have shown promise as novel therapeutics for colorectal disease by directly inhibiting the VCAM-1/α_4_β_1_ interaction. While therapeutic antibodies are superior in terms of target selectivity, SMDs are virtually non-immunogenic, granting them greater tolerability in intermittent and cyclic treatment regimens [97]. Carotegrast methyl (AJM300) is an SMD inhibitor of the α_4_ subunit that has recently received approval in Japan following its success in clinical trials [98, 99]. Clinical studies by Yoshimura et al [100] and Matsuoka et al [97] supported the safety and efficacy of AJM300 for active UC patients who had previously exhibited inadequate response or intolerance to mesalamine or corticosteroids. Notably, AJM300 was specifically formulated for oral administration. This bestows it a significant advantage compared to most other drug candidates targeting the VCAM-1/α_4_β_1_ interaction, which are generally administered intravenously. The increased incidence of progressive multifocal encephalopathy associated with natalizumab has so far not been as evident in AJM300 studies. However, the shared mechanism of α_4_ blockade would suggest this is still a risk. Although AJM300 has been approved for use in moderate UC, further studies should be conducted to properly clarify the association between integrin inhibition and the development of progressive multifocal encephalopathy.

### Anti-VCAM-1 antibodies

Antibodies directly targeting VCAM-1 present an attractive strategy for the selective blockade of VCAM-1-mediated cell recruitment during intestinal disease. The therapeutic potential of anti-VCAM-1 antibodies has been investigated in various experimental inflammatory disease models, including atherosclerosis [101], asthma [102], and rheumatoid arthritis [103]. In a study characterising the roles of ICAM-1 and VCAM-1 during colitis, Sans et al [50] highlighted the therapeutic potential of VCAM-1 antibody blockade for preventing pathological leukocyte infiltration and associated intestinal inflammation. The authors found that ICAM-1 and, to a greater degree, VCAM-1 antibody blockade significantly attenuated leukocyte adhesion to colonic venules in experimental rat models of 2,4,6-trinitrobenzene sulphonic acid (TNBS)-induced colitis. Following these experiments, long-term treatment of colitic animals with 5F10 anti-VCAM-1 mAb was shown to significantly reduce inflammatory cell infiltrate, macroscopic tissue damage, and colitis symptoms. Interestingly, the authors found that simultaneous ICAM-1/VCAM-1 blockade did not further inhibit leukocyte adhesion compared to VCAM-1 blockade alone, implicating VCAM-1 as an optimal adhesion target for selective inhibition of colitis-associated leukocyte recruitment [50]. The superior anti-colitic effects of VCAM-1 antibody blockade compared to other endothelial CAM targets were later corroborated by Soriano et al [55], who demonstrated that chronic treatment with MK1.91 anti-VCAM-1 mAbs, but not with ICAM-1 mAb or MAdCAM-1 mAbs, significantly improved dextran sodium sulphate (DSS)-induced murine colitis. Moreover, the authors commented that the specific inhibition of a particular endothelial CAM may be preferable for more selective pharmacological activity and minimal effects on normal physiological trafficking.

However, the superiority of VCAM-1 to other endothelial CAMs was brought into contention by later studies. Watanabe et al [80] demonstrated that 429 anti-VCAM-1 mAb decreased splenic lymphocyte adhesion in colonic venules. The authors also noticed that combined blockade of VCAM- and MAdCAM-1 further inhibited cell adhesion compared to separate inhibition. In addition to their anti-inflammatory activity against IBD, anti-VCAM-1 antibodies may also pose a therapeutic benefit in preventing tumour proliferation and metastasis in CRC. Chu et al [104] developed domain-based bispecific antibodies to VCAM-1 and glycoprotein non-metastatic melanoma protein B (GPNMB) to test their efficacy in the selective killing of cancer cells. The authors demonstrated that the antibodies were able to specifically kill VCAM-1 and GPNMB-expressing lines, and non-specific killing was wholly avoided at all but the highest test concentration [104]. The promising results of this study, combined with the established involvement of VCAM-1 in CRC progression, warrant investigations into the therapeutic effects of anti-VCAM-1 antibodies on specific colon cancer lines [51]. The high specificity of antibody-based immunotherapies makes them an attractive option for targeted treatment of cancer and inflammation. Therefore, therapeutic anti-VCAM-1 antibodies remain a subject of interest within the scope of both IBD and CRC.

### VCAM-1 antisense oligonucleotides

An alternative strategy for directly inhibiting VCAM-1-mediated cell trafficking during colonic disease is specific VCAM-1 knockdown by antisense oligonucleotides (ASOs). Antisense gene suppression using oligonucleotides has been demonstrated to selectively inhibit the expression of different endothelial CAMs (including ICAM-1, VCAM-1, ELAM-1, and E-selectin) on inflamed endothelia *in vitro* [105, 106]. ASOs therefore present a viable means of selectively blocking specific routes of cell recruitment to effectively reduce pathological leukocyte infiltration and tumour cell extravasation with minimal off-target effects on normal cell trafficking. As a proof-of-concept, Rijcken et al [79] developed 2’-O-methoxyethyl chimeric ASOs directed against VCAM-1 (ISIS 18155) and ICAM-1 (ISIS 18111) and evaluated their ability to directly inhibit leukocyte adherence and infiltration during intestinal inflammation. The authors demonstrated that by suppressing VCAM-1 and ICAM-1 expression in submucosal and mesenteric venules, ASOs were able to dose-dependently attenuate histological and macroscopic inflammation in colitic rat models [79]. In a head-to-head comparison of the ICAM-1 and VCAM-1 ASOs in terms of therapeutic efficacy, the authors noted that while ICAM-1 was more effective at lower dosage concentrations, VCAM-1 represented a far more specific target for intestinal inflammation, similar to findings observed in tests of therapeutic anti-VCAM-1 antibodies. These results suggest that regarding the balance of drug efficacy and safety, VCAM-1 may present a more suitable drug target for specificity when treating colitic lesions.

Further research into the anti-colitic effects of VCAM-1 antisense therapy beyond Rijcken et al.’s study has apparently been set aside in favour of ASO candidates targeting other endothelial CAMs. In particular, the ICAM-1-directed ASO, alicaforsen (ISIS 2302), has been thoroughly investigated as a novel therapy for CD and UC in clinical trials [107, 108]. Results have been somewhat divided, with several studies observing no significant differences between control and placebo groups in clinical remission at the primary endpoint. However, several of these studies still demonstrate a modest and durable improvement of intestinal disease symptoms of alicaforsen, indicating the possible efficacy of the drug mechanism. The promise of alicaforsen as a drug candidate for IBD suggests a similar utility of VCAM-1 ASOs in colitis, which may even be better with the therapeutic blockade of an alternative adhesion molecule. However, a suitable VCAM-1 ASO candidate is yet to advance beyond *in vivo* testing. Despite the promise of other ASOs in cancer models, VCAM-1 ASOs have yet to be investigated in experimental models of colorectal cancer. Regardless of their decreased use in more recent years, VCAM-1-targeted ASOs could still present a viable drug strategy for the targeted treatment of colonic diseases.

## VCAM-1-targeted systems for visualisation and drug delivery

So far, this review has discussed the application of VCAM-1-directed therapeutics for the selective inhibition of migration-based mechanisms underlying inflammatory cell infiltration and cancer cell metastasis. However, a fair share of research attention has also been devoted to the potential utility of VCAM-1 as a cell surface receptor for the active targeting of functionalised nanosystems.

VCAM-1-antagonising peptides and antibodies have been widely utilised as targeting moieties for visualising inflammation and delivering anti-inflammatory agents in disease pathologies involving endothelial dysfunction, particularly atherosclerosis [109, 110]. Similarly, VCAM-1-functionalised nanosystems have also proven promising as a potential platform for cancer theranostics [111, 112]. The upregulation of VCAM-1 on inflamed colonic vessels and colorectal tumour vasculature, coupled with its involvement in immune cell infiltration and cancer cell metastasis, has highlighted it as an attractive candidate for receptor-mediated imaging and drug delivery systems in colorectal disease. However, research attention has been divided between different endothelial CAM receptor targets, meaning VCAM-1-targeted systems have been somewhat overshadowed by their ICAM-1- [113] and MAdCAM-1-targeted [114] counterparts. Although there is no definitive consensus as to which endothelial CAM ligand is most suitable for colorectal disease treatment, the restriction of MAdCAM-1 expression to gut-associated epithelia marks it as a preferable target for minimising extracolonic accumulation and systemic toxicity. However, the localised expression of VCAM-1 in extracolonic sites of carcinogenesis and inflammation raises the possibility of identifying and treating IBD comorbidities and secondary tumours of metastatic CRC. To fully exploit the potential of this functional versatility, further studies characterising the properties and effects of VCAM-1-conjugated nanosystems in experimental colonic disease models are warranted.

### VCAM-1-targeted systems for visualising inflammation

VCAM-1-targeted optical nanoprobes that can selectively image the activated mesenteric microvasculature associated with pathological cell extravasation may become a valuable tool for detecting and quantitating colorectal disease activity. Complementary to conventional endoscopy assessments, non-invasive cross-sectional imaging and ultrasonography techniques have proven advantageous in evaluating the extent and severity of colitic lesions during IBD [115]. For CRC diagnostics, radiologic imaging is also the principal means of initial disease staging and subsequent surveillance for disease recurrence [116]. In the wake of recent advances in non-invasive imaging technologies, receptor-functionalised ‘nanobeacons’ that can specifically bind and visualise biomarker ligands on the intestinal mucosa are beginning to gain research interest as a possible imaging modality for detecting and quantifying disease activity during colonic disease [117]. In particular, the localised expression of VCAM-1 to areas of inflammation can be exploited to achieve specific visualisation of colitic lesions and colorectal tumours by labelling the peripheral activated vasculature associated with these disease sites. VCAM-1-targeted probes have already shown promise in experimental models of cardiovascular [109, 118, 119] and neurologic [120] diseases. Drawing from this rationale, studies have emerged to investigate the efficacy of VCAM-1-targeted probes for visualising colitic lesions during IBD [121–123]. VCAM-1-based visualisation is yet to be explored in the context of CRC, but the promise of anti-VCAM-1 antibodies and VCAM-1-conjugated systems in other cancer models implies the likelihood of favourable outcomes for colorectal tumour visualisation [104, 111, 112].

#### anti-VCAM-1 antibodies

Radiolabelled anti-VCAM-1 mAbs have been investigated as potential scintigraphy tracers for detecting and evaluating colitic lesions *in vivo* [121, 122]. Sans et al [121] first demonstrated the diagnostic capability of anti-VCAM-1 mAbs for assessing colonic inflammatory damage by performing scintigraphic and biodistribution studies of intravenously administered iodine-123 (^123^I)-labelled 5F10 probes in rat models of TNBS-induced colitis. The authors demonstrated that specific binding and uptake of anti-VCAM-1 mAb probes were significantly higher in colitic rats compared to control animals, constructing scintigraphic images that correlated to increased expression of VCAM-1 in the inflamed colon. Aside from the colon, scintigraphic uptake of anti-VCAM-1 mAb also reached statistical significance in the liver and spleen, but extracolonic accumulation did not differ between colitic and control animals [121]. These results were corroborated by a later study from Liu et al [122], which evaluated anti-VCAM-1 single-chain variable fragments (scFvs) radiolabelled with technetium-99 m (^99m^Tc) as scintigraphic markers for IBD diagnosis and evaluation. Single photon emission computed tomography (SPECT) and computed tomography (CT) imaging confirmed that anti-VCAM-1 mAb probe uptake was significantly enhanced in the distal colons of colitic rabbits compared to corresponding controls, which correlated to heightened VCAM-1 expression in immunohistochemical samples of colonic tissues [122]. The cumulative findings of these two studies support the diagnostic mechanism of targeted antibodies for specifically binding VCAM-1 to precisely evaluate the location and extent of colonic inflammation during IBD.

Within the research scope of colonic imaging techniques, radiolabelled anti-VCAM-1 antibodies have so far only been investigated and employed for evaluating IBD. However, the selective involvement of VCAM-1 across several other colonic and extracolonic disease pathologies suggests a possible functional versatility of anti-VCAM-1 antibodies that is yet to be fully explored. The upregulation of VCAM-1 on colorectal tumour tissues compared to normal colonic tissues implies the potential utility of anti-VCAM-1 antibodies for visualising not only sites of colitis but also malignant polyps and neoplastic lesions during CRC. Furthermore, the extracolonic accumulation of radiolabelled anti-VCAM-1 antibodies observed from *in vivo* biodistribution studies [121], while traditionally considered a limitation in imaging specificity, may present an intriguing possibility for screening colorectal comorbidities involving dysregulated VCAM-1 activity. For instance, concentrated regions of aberrant VCAM-1 expression, either on inflamed endothelia or on tumour surfaces, could potentially detect IBD-associated inflammatory conditions or sites of CRC metastasis when screening for colorectal disease. The most distinct advantage of anti-VCAM-1 antibodies is their specificity; thus, a great deal of research attention has shifted to their application as targeting moieties in conjugated nanocarrier systems. Though this has somewhat overshadowed the use of immunoblocking antibodies as therapeutic agents for IBD and CRC, studies into anti-VCAM-1 antibody mechanisms and efficacy still provide significant insights into cell adhesion-targeting strategies in colonic diseases.

#### VCAM-1-conjugated microbubbles

Contrast-enhanced ultrasound imaging using VCAM-1-conjugated microbubbles has also been studied as a potential imaging modality for visualising colonic inflammation in IBD. Microbubbles are gas-containing microspheres that have been widely employed as contrast enhancers for ultrasound imaging and carrier agents for targeted drug delivery [124]. Yang et al [125] supported the utility of VCAM-1 as a targeting ligand for microbubble systems by demonstrating that VCAM-1 mAb-conjugated microbubbles significantly increased adhesion to LPS-activated HUVECs *in vitro* compared to non-targeted control microbubbles. Several studies have developed VCAM-1-targeted microbubble systems for visualising atherosclerotic plaques [126, 127]; however, these systems have been underutilised in the context of IBD. A study by Tlaxca et al [123] demonstrated that lipid-shelled microbubbles functionalised with anti-VCAM-1 antibodies were able to selectively bind to the inflamed mesenteric endothelium for ultrasound-based molecular imaging *in vivo* using CD-like TNFΔARE mouse models. Punctate contrast patterns observed by ultrasound imaging demonstrated the selective intravascular binding of the targeted microbubbles to areas of aberrant VCAM-1 expression, representing inflamed colonic tissues [123]. These results support the usefulness of VCAM-1 functionalisation for visualising inflammation while preventing binding to adjacent ‘bystander’ tissues, implying a molecular specificity superior to conventional imaging techniques.

Although investigated to a lesser extent compared to their utility in IBD, VCAM-1-conjugated microbubbles have also demonstrated a potential application for ultrasound imaging in CRC. Microbubble systems targeted to molecular markers on angiogenic endothelia have been proposed as an intriguing diagnostic platform for detecting and monitoring tumour neovascularisation during cancer, and VCAM-1 serves as a cancer-indicative biomarker for tumour-associated microvessels [128]. The micron-scale diameter of microbubbles restricts them to the intravascular compartment, marking them as practical blood pool agents for functional imaging of angiogenesis by visualising blood flow and perfusion at tumour tissues [129]. In a study testing the diagnostic capabilities of ligand-decorated microbubbles *in vivo*, Unnikrishnan et al [130] employed VCAM-1 peptide-conjugated microbubbles for ultrasound imaging of tumour vasculature in MC38 colon adenocarcinoma tumour-bearing mice. The authors demonstrated that their VCAM-1-targeted microbubble formulation was effectively and selectively retained in the tumour vasculature with significantly lower retention in the contralateral leg muscle. These results denoted a superior selectivity to non-targeted control microbubbles, which did not accumulate in the tumour vasculature [130]. The improved specificity of VCAM-1-conjugated microbubble systems thus presents a functional and convenient platform for grading CRC upon presentation and evaluating the effectiveness of anti-angiogenic therapies after initial diagnosis.

### VCAM-1-targeted systems for targeted drug delivery

Receptor-mediated imaging and drug delivery systems are certainly not a foreign concept in the context of colorectal disease research, and VCAM-1-conjugated nanocarriers represent a viable avenue of study. Membrane-targeted nanosystems are generally considered a very attractive strategy for selective drug accumulation in the GI tract, as they can precisely locate and exploit the inflamed colonic endothelium for enhanced drug permeability and retention [131]. Furthermore, targeted drug delivery into the colon is highly desirable for reducing systemic toxicity, especially for highly toxic drugs such as chemotherapeutics. Most importantly, colon-specific delivery systems need to be capable of protecting the drug *en route* to the colon to avoid premature stomach absorption or degradation. Some conventional examples of colon-targeted drug delivery systems include prodrugs [132], pH- [133] and time-dependent release systems [134], and bacteria-responsive [135] carrier systems. However, the unpredictable nature of the dysregulated gut microbiome during colonic disease means that digestive parameters, such as transit time and physiological pH, frequently vary between patients and are, therefore, virtually impossible to account for in drug design. Receptor-functionalised nanocarrier systems targeting endothelial biomarkers such as VCAM-1 aim to circumvent these challenges. VCAM-1-conjugated nanocarriers have previously been shown to improve the water solubility of anti-inflammatory drugs to enhance their bioavailability and drug delivery. Naturally, a range of VCAM-1-conjugated nanocarriers have been designed and investigated as potential platforms for treating IBD and CRC. While this remains a highly novel study area, VCAM-1-targeted nanocarriers constitute an area of keen interest for targeted colon treatment.

#### Mesenchymal stem cells

In recent years, studies have put forward cell surface coating of mesenchymal stem cells (MSCs) with anti-VCAM-1 mAbs to enhance cell targeting to sites of colonic tissue injury during colorectal disease. MSCs are pluripotent non-haematopoietic stem cells that have been shown to exert immunomodulatory effects by secreting trophic and anti-inflammatory factors such as cytokines, chemokines, and growth factors [136]. Previous studies have demonstrated the capabilities of MSCs for reducing chronic colonic inflammation and regulating gut microbiome dysbiosis, suggesting a possible utility for the treatment and prevention of both IBD and colitis-associated CRC [137]. In recent years, the therapeutic potential of functionalising MSCs with targeting ligands to enhance cell homing to diseased tissues has emerged as a particular area of research interest. A comparative research study by Ko et al [138] demonstrated that anti-VCAM-1 antibody–coated MSCs exhibited higher rates of delivery efficiency to the mesenteric lymph nodes and inflamed colon compared to uncoated MSCs, isotype antibody–coated MSCs, and anti-MAdCAM-1 antibody–coated MSCs. Interestingly, VCAM-1 antibody–coated MSCs also demonstrated significantly enhanced therapeutic and suppressive capabilities compared to all other MSC candidates. This observation could be attributed in part to the improved delivery mechanism but, also, as hypothesised by the authors, to synergistic immunomodulatory effects released by anti-VCAM-1 antibodies [138].

#### Polymeric nanoparticles

Polymeric nanoparticles (NPs) have also been explored as a potential platform for VCAM-1-targeted drug delivery systems in colorectal disease. Compared to conventional colon-targeted drugs, polymeric NP formulations—including poly(lactide-co-glycolide) (PLGA), poly(lactic acid) (PLA), poly(ethylene glycol) (PEG), chitosan, and gelatin—exhibit advantageous features such as efficient drug encapsulation, improved durability to enzymatic and microbial degradation, and increased mucosal adhesion and absorption within the GI tract [139]. Several novel polymeric NP systems have exhibited promising results in experimental models of IBD and CRC, unveiling their potential as therapeutic nanocarriers for early diagnosis and efficient treatment of colonic diseases [140, 141] Sakhalkar et al [142] first explored the benefits of NP-antibody coupling for targeted drug delivery by conjugating biodegradable PEG-PLA block polymer NPs with different endothelial CAM antibody ligands, including VCAM-1, and comparing their adhesion to inflamed endothelia. The authors found that the NPs selectively accumulated at cytokine- and trauma-stimulated endothelia relative to non-inflamed endothelia both *in vitro* and *in vivo* [142]. As a continuation of this study, Sakhalkar et al [143] then tested the adhesive capacity of anti-VCAM-1 mAb-conjugated PEG-PLA NPs to the colonic vasculature in colitic mouse models. The authors demonstrated that their VCAM-1-targeted NPs exhibited more specific and augmented accumulation on colonic venules of colitic mice compared to healthy controls [143]. Following these promising results, the next step will be to assess the therapeutic effectiveness of drug-loaded VCAM-1-conjugated polymeric NPs in experimental models of IBD.

#### Microbubbles

Similar to their utility as optical nanoprobes for visualising diseased colorectal tissues, VCAM-1-functionalised microbubbles may also present a potential strategy for targeted drug delivery in colorectal disease. Ultrasound-mediated microbubble delivery, in which microbubbles transport a drug or gene to desired tissues for destruction by targeted ultrasound irradiation, has been proposed as an innovative technique for non-invasive, site-specific delivery of bioactive materials [144]. The distinct advantage of microbubbles as a nanocarrier platform is that the ultrasonic field can be focused precisely on tissues and organs of interest, thereby minimising the risk of undesirable off-target effects [145]. Conjugating microbubble vehicles with targeting antibodies is a possible strategy for improving the specificity of microbubble systems, and the localisation of VCAM-1 to disease-associated vasculature marks it as a highly attractive candidate ligand for this purpose. Tlaxca et al [123] found that once intravenously administered in colitic TNFΔARE mice, VCAM-1 and MAdCAM-1-targeted microbubbles selectively accumulated in the lower abdominal cavity for site-specific delivery of luciferase plasmid. Furthermore, microbubble accumulation was significantly higher in the disease model compared to wild-type controls, which suggested that elevated VCAM-1 and MAdCAM-1 expression following colitis induction mediated the inflammation-dependent binding and drug delivery of the targeted microbubble system [123]. From the results of this study, VCAM-1-conjugated microbubbles seem to present a practical and intriguing platform for targeted colon treatment.

## Perspectives and conclusions

The involvement of VCAM-1 in both inflammatory cell infiltration and cancer cell metastasis bestows it a remarkable functional versatility as a drug target for a wide range of diseases. In the context of colorectal disease research, the role of VCAM-1-mediated cell-to-cell interactions in both IBD and CRC creates an intriguing possibility for colon-directed, VCAM-1-antagonising drug systems with dual applications against both pathologies. These synergistic anti-inflammatory and anti-cancer effects are well worthy of attention, considering that IBD patients have a significant predisposition for developing colitis-associated CRC. With this in mind, the notion of a VCAM-1-directed, anti-colitic drug with additional chemopreventive effects could substantially improve disease maintenance and prognosis for IBD patients. Several anti-VCAM-1 drug candidates have already been investigated separately in IBD and CRC models. However, very few studies have explicitly commented on the possibility of dual functionality across both diseases. This is primarily because it is so challenging to create an experimental model that can faithfully and consistently recapitulate the progression of IBD to CRC as observed in humans. At present, the most substantive evidence can be gained from colitis-associated CRC animal models, particularly the murine azoxymethane (AOM)/DSS-induced model [147]. Furthermore, studies documenting the anti-atherosclerotic effects of VCAM-1 inhibiting therapeutics in cardiovascular disease models bring to light the possibility of anti-colitic drugs with auxiliary effects on comorbid atherosclerotic lesions [41]. While well established, the relationships between related colorectal and cardiovascular pathologies can be highly complex, which warrants the need for more comprehensive studies and investigations into the multi-functional capacity of VCAM-1-directed therapies.

VCAM-1 also functions as a very interesting targeting moiety for medical imaging and drug delivery systems in colonic disease. The inherent immunoblocking activity of anti-VCAM-1 agents means that anti-VCAM-1-peptide and antibody-conjugated nanosystems may be able to synergistically inhibit pathological cell migration processes while specifically delivering drugs to sites of inflammation. This review describes several existing studies of VCAM-1-functionalised diagnostic and therapeutic nanosystems that demonstrate the enhanced drug accumulation and therapeutic efficacy of conjugating vehicles with anti-VCAM-1 vectors. However, it remains undetermined whether these observations can be attributed purely to more efficient drug delivery or may also be a consequence of the inherent therapeutic effects of certain VCAM-1-binding agents. The continual improvement of enteric microencapsulation and coating techniques for oral drug formulations has prompted a notable shift towards oral administration in the rational drug design of colon-targeted drug systems. Despite this trend, existing VCAM-1-functionalised nanosystems for visualising and treating colonic diseases have only been administered intravenously. This apparent hesitation towards developing oral VCAM-1-conjugated nanoformulations is likely due to the inherent challenges of oral delivery for reaching endothelial drug targets compared to conventional vascular drug delivery. Naturally, one of the most significant obstacles in adapting VCAM-1-targeted vehicles for oral delivery is designing a carrier system that is capable of not only surviving the GI environment but also crossing the gut-blood barrier to bind target VCAM-1 on the mesenteric endothelium. Although not yet tested in the context of colorectal disease, VCAM-1-functionalised lipid nanoemulsions have previously shown promise for oral delivery to sites of endothelial inflammation [148]. As such, VCAM-1-directed nanosystems still present an attractive avenue for designing and developing inflammation-targeted colorectal disease therapeutics.

## Data Availability

No datasets were generated or analysed during the current study.

## Abbreviations

*AJM300*: Carotegrast methyl
*AOM*: Azoxymethane
*ASO*: Antisense oligonucleotide
*CAM*: Cell adhesion molecule
*CD*: Crohn’s disease
*CRC*: Colorectal cancer
*DSS*: Dextran sodium sulphate
*ERK*: Extracellular signal–related kinase
*FAK*: Focal adhesion kinase
*GI*: Gastrointestinal disease
*GPNMB*: Glycoprotein non-metastatic melanoma protein B
^*123*^*I*: Iodine-123
*IBD*: Inflammatory bowel disease
*ICAM*: Intercellular adhesion molecule
*Ig*: Immunoglobulin
*LPS*: Lipopolysaccharide
*mAb*: Monoclonal antibody
*MAdCAM*: Mucosal addressin cell adhesion molecule
*MAP*: Mitogen-associated protein
*MSC*: Mesenchymal stem cell
*NF-κB*: Cancer factor-κB
*NP*: Nanoparticle
*PEG*: Poly(ethylene glycol)
*PLA*: Poly(lactic acid)
*PLGA*: Poly(lactide-co-glycolide)
*ROS*: Reactive oxygen species
*scFv*: Single-chain variable fragment
*SMD*: Small molecule drug
*SPECT*: Single photon emission computed tomography
^*99*^*mTc*: technetium-99m
*TNBS*: 2,4,6-trinitrobenzene sulphonic acid
*TNF-α*: tumour necrosis factor-α
*VCAM*: vascular cell adhesion molecule
